# Symptoms of somatization as a rapid screening tool for mitochondrial dysfunction in depression

**DOI:** 10.1186/1751-0759-2-7

**Published:** 2008-02-22

**Authors:** Ann Gardner, Richard G Boles

**Affiliations:** 1 Karolinska Institutet, Department of Clinical Neuroscience, Section of Psychiatry, Karolinska University Hospital Huddinge, SE-141 86 Stockholm, Sweden; 2 Division of Medical Genetics and the Saban Research Institute, Childrens Hospital Los Angeles, CA 90027, USA; 3 Department of Pediatrics, Keck School of Medicine, University of Southern California, Los Angeles, CA 90027, USA

## Abstract

**Aims:**

Somatic symptomatology is common in depression, and is often attributed to the Freudian-inspired concept of "somatization". While the same somatic symptoms and depression are common in mitochondrial disease, in cases with concurrent mood symptoms the diagnosis of a mitochondrial disorder and related therapy are typically delayed for many years. A short screening tool that can identify patients with depression at high risk for having underlying mitochondrial dysfunction is presented.

**Methods:**

Six items of the Karolinska Scales of Personality (KSP) were found to differentiate among 21 chronically-depressed Swedish subjects with low versus normal muscle ATP production rates. A screening tool consisting of the six KSP questions was validated in the relatives of American genetics clinic patients, including in 24 matrilineal relatives in families with maternally inherited mitochondrial disease and in 30 control relatives.

**Results:**

Among the depressed Swedish patients, the screening tool was positive in 13/14 with low and 1/7 with normal mitochondrial function (P = 0.0003). Applied to the American relatives of patients, the screening tool was positive in 13/24 matrilineal relatives and in 1/30 control relatives (P = 2 × 10^-5^).

**Conclusion:**

Our preliminary data suggest that a small number of specific somatic-related questions can be constructed into a valid screening tool for cases at high risk for having a component of energy metabolism in their pathogenesis.

## Findings

Multiple medically-unexplained somatic complaints, usually associated with mood symptoms, is currently designated as "somatization" under the Diagnostic and Statistical Manual (DSM) system used to classify mental illness. This phenomenon has been observed by physicians for at least 3000 years and was known as "hysteria" until the previous DSM designation of "Briquet's syndrome". The French physician Briquet, in his 1859 treatise on hysteria, described mood as well as a long list of somatic symptoms among his 430 patients, including migraine, abdominal pain, muscle pain, palpitations, restlessness, hyperesthesias, anesthesias and fatigue [1]. While Briquet assumed that his patients' symptomatology had a biological basis, many mental health providers today practice under the assumption that prominent somatic symptomology among depressed individuals are of psychic origin, a theory often attributed to the work of Sigmund Freud.

However, in 1895, Freud and Breuer in their classical study of hysteria [2] actually postulated the presence of underlying genetic-biological factors in their patients as exemplified by the comments by Freud " [she] was undoubtedly a person with a severe neuropathic heredity. To accomplish hysteria without a disposition of this kind would most likely be impossible" (page 93), and "...somatic pain was not created by the neurosis, but simply used, heightened and maintained by it" (page 177). The popular concept of the symbolic conversion of psychic conflicts into somatic symptoms by the unconscious mind was presented by Freud and Breuer in order to explain the specific somatic manifestations reported, not necessarily their initial pathogenesis.

Unfortunately, considering the high prevalence and associated disability of depression with somatization, there has been little advancement in our understanding of its pathogenesis since the time of Freud. As the same somatic and mood symptoms are common in patients with mitochondrial disorders [3-8], recently-described genetic conditions that result from a decreased production of adenosine triphosphate (ATP, cellular energy), we have hypothesized that a relative cellular energy depletion can serve as an important risk factor predisposing some individuals towards the development of both somatic symptomatology and depression [9].

There is substantial overlap between patients identified with somatization and with mitochondrial disorders. In the latter conditions, symptoms typically wax and wane, and involve different tissues and body locations at different times, presumably in accordance with local energy supply and demand. These and other factors often lead the patient's symptoms to be dismissed as being "functional" or "psychosomatic", delaying typically by many years the specific testing that establishes the diagnosis of a mitochondrial disorder in patients with concurrent mood symptoms [4,8].

Recently, we reported on *in vitro *muscle ATP production rates using α-ketoglutarate as substrate in 21 adult Swedish subjects with longstanding major depression [10]. Consistent with previous findings [11], almost one-half of our chronically-depressed subjects exhibited very high levels of somatic complaints on self-reported scales indicating somatization. Essentially every one of those "somatizing" depressed subjects demonstrated a muscle ATP production rate below that measured in 10 healthy controls [10]. However, while supporting our hypothesis regarding a mitochondrial component in the pathogenesis of depression with somatization, our findings are of limited immediate clinical utility since the modalities that were used were 30 items from the Karolinska Scales of Personality (KSP) and an open muscle biopsy.

Inspired by the World Health Organization six-item, 5-point response format, self-report scale for the screening of adult Attention Deficit Hyperactivity Disorder (ADHD) [12], we have herein taken the first steps towards the development of a screening tool that rapidly can identify those individuals that are most likely to have mitochondrial dysfunction, and hence most likely to benefit from related therapies.

We re-evaluated our data [10] from the 21 adult subjects from Sweden with longstanding major depression and audiological disease (hearing loss or tinnitus) mentioned above in terms of the responses to individual KSP items in subjects with low *versus *with normal ATP production rates.

In order to validate our screening instrument, English translations [13] of the six KSP items with the highest degrees of association with mitochondrial function in the above group were added to a larger five-page questionnaire that was self-completed by a second set of subjects. Mothers were recruited from the Genetics clinics at Childrens Hospital Los Angeles; mothers of mitochondrial patients were asked to recruit specific relatives as well. The "mitochondrial group" consisted of 24 subjects: 17 mothers and 7 maternal aunts of patients with maternally inherited mitochondrial disease. Since the mitochondrial DNA (mtDNA) is maternally inherited intact from a mother to all of her children without recombination or paternal component, those relatives have the same mtDNA sequence as the patients, and presumably some degree of energy depletion. Mitochondrial disease was defined by the established Nijmegen criteria (at the level of "probable" or "definite") [14], and maternal inheritance was defined by our quantitative pedigree analysis method [15]. The "control group" consisted of 30 subjects: 6 paternal aunts and 6 aunts-in-law (uncles' wives) of the same families, thus having different mtDNA sequences than our patients, as well as 18 mothers of clinic patients with autosomal recessive metabolic disorders.

Three of the 15 KSP scales, Somatic Anxiety, Muscular Tension and Psychasthenia, inquire regarding somatic symptomology or reactions to that symptomology [10,13], and were the focus of this study. The KSP has four possible choices for each item: "Applies Completely" (score 4), "Applies Rather Well" (score 3), "Does Not Apply Well" (score 2) and "Does Not Apply at All" (score 1). Since some degree of somatic symptoms are nearly universal, yet in our experience are highly exaggerated in patients with mitochondrial disorders, we evaluated the items based upon the maximum score of 4 ("Applies Completely") versus all other scores.

Among the American relatives, the presence of "depression" was defined as a probable diagnosis of major depressive disorder based upon meeting the DSM Version-IV-TR criteria for that condition from questionnaire data, or the relative had been given a diagnosis of "depression" and treated for such by a medical or mental health professional.

**Figure 1:**
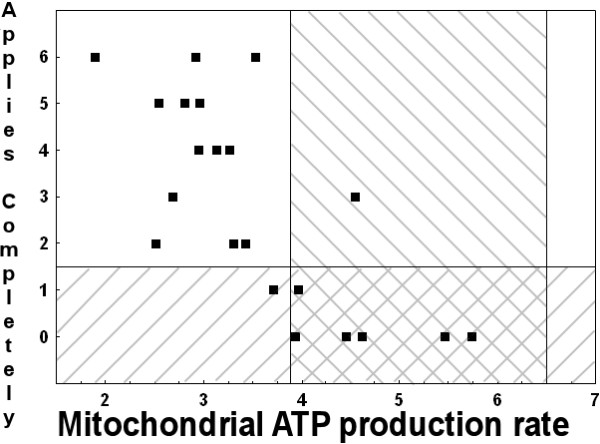
**Number of "Applies Completely" responses to the six KSP items in Table 1**. The hatched area within the left-sloping diagonal bars corresponds to the proposed "normal" range of answering zero or one of the six items as "Applies Completely" (Y axis). The hatched area within the right-sloping diagonal bars corresponds to the range of muscle ATP production rates measured in 10 healthy controls using α-ketoglutarate as substrate (3.9 – 6.5 mmol ATP min^-1 ^kg^-1 ^muscle). α-ketoglutarate predominately stimulates respiration through complex I of the electron transport chain. Complex I deficiency is the most common enzymatic deficiency in the mitochondrial disorders [24].

In our 21 depressed subjects from Sweden, a score of 4 was found to be statistically more common (P < 0.05) in 10 of the 30 somatization-related KSP items [10] among the subjects with low ATP production rates versus among the subjects with normal ATP production rates. In contrast, a score of 4 was not found to be statistically more common among subjects with normal ATP production rates for any of the 30 items. For six of the 10 items (Table 1), a score of 4 was selected by at least eight of the 14 subjects with low ATP production rates, and by no more than one of the seven subjects with normal ATP production rates. Thirteen of the 14 subjects with ATP production rates below the normal range answered at least two of those six items as "Applies Completely" (sensitivity = 93%), versus only 1 of 7 subjects with ATP production rates within our control range (specificity = 86%, chi-square P = 0.0003) (Figure 1).

**Table 1:** Brief screening test developed from six selected items of the Karolinska Scales of Personality

**KSP question answered by participants**	**KSP scale containing the question**	**KSP Item #**	**Low vs. normal ATP production rate, Swedish subjects**	**Mitochondrial vs. control groups, American subjects**
My heart sometimes beats hard or irregularly for no real reason.	Somatic Anxiety	34	10/14 vs. 0/7 P = 0.003	13/24 vs. 0/30 P = 3.7 × 10^-6^
I often have aches in my shoulders and in the back of my neck.	Muscular Tension	4	9/14 vs. 1/7 P = 0.031	12/24 vs. 1/30 P = 6.7 × 10^-5^
My body often feels stiff and tense.	Muscular Tension	33	9/14 vs. 1/7 P = 0.031	5/24 vs. 0/30 P = 0.013
I think I must economize my energy.	Psychasthenia	40	8/14 vs. 0/7 P = 0.015	6/24 vs. 1/30 P = 0.025
In order to get something done I have to spend more energy than most others.	Psychasthenia	53	9/14 vs. 1/7 P = 0.031	6/24 vs. 1/30 P = 0.025
I feel easily pressured when I am urged to speed up.	Psychasthenia	93	10/14 vs. 1/7 P = 0.014	2/24 vs. 1/30 Non-significant (P = 0.42)

The numbering of items refers to the 135-items KSP questionnaire. P-values refer to chi-square or Fisher exact test values in participants answering as "Applies Completely".

In the American relatives of subjects, five of the six KSP items listed in Table 1 were answered as "Applies Completely" statistically more often in the mitochondrial group versus in the subjects in the control group (Table 1, right-hand column). At least two of the six-KSP items listed in Table 1 were answered as "Applies Completely" in 13/24 of the mitochondrial group (10/17 mothers and 3/7 maternal aunts) and in 1/30 of the control group (0/6 paternal aunts, 0/6 aunts-in-law and 1/18 mothers of children with recessive disorders) (P = 2 × 10^-5^). This corresponds to 54% sensitivity and 97% specificity. Eleven of the 24 (46%) relatives in the mitochondrial group had a lifetime diagnosis of depression, versus 3 of 28 (11%) control relatives (P = 2 × 10^-5^). There was no correlation between the presence or absence of depression and the KSP screening results.

Among our Swedish subjects with lower levels of mitochondrial function, the choice of the extreme "Applies Completely" response may reflect either more intense symptomatology and/or the tendency of a more dramatic account of the state, a "hysterical" disposition. The lower sensitivity of our proposed six-question screening battery in the American versus in the Swedish subjects may be due to a lower degree of symptomatology in the former, predominately reproductive-aged (mean 43 ± 9 years, median = 41 years) relatives of patients versus in the our older patients (mean 49 ± 9 years, median = 51 years, Students t-test P = 0.021 for the mean age difference between these groups) who themselves suffer from chronic mental illness. Applying the American data in a highly-selected population whereas 67% have some degree of energy depletion (equal to that in our Swedish cohort), our data corresponds to a 93% positive predictive value and a 72% negative predictive value for the screening battery, with a positive response defined as 2 to 6 questions answered as "Applies Completely". In a hypothetical population of lesser acuity whereas only 10% have some degree of energy depletion, our data corresponds to a 64% positive predictive value and a 95% negative predictive value.

In the functional disorder of migraine, which demonstrates strong co-morbidity with depression [16], co-enzyme Q10 [17] and riboflavin [18], a component and a precursor of a component of the mitochondrial respiratory chain, have shown efficacy in double-blind, placebo-controlled clinical trials. Creatine, a source of stored energy for brain, has recently been reported to be beneficial in unipolar depression resistant to pharmacological treatment [19]. These "mitochondrial-directed" therapies have demonstrated efficacy in patients with mitochondrial disorders [20-22]. In our anecdotal observations with the same therapies, and lipoic acid [20], improvement in somatic symptomatology, and possibly in depression itself, was noted in over half of the depressed subjects. The potential for effective therapy underscores the need to screen depressed patients with somatic symptoms for those that might benefit from additional mitochondrial-related investigations (especially quantitative urine organic acids while symptomatic) and/or therapies.

We propose that energy depletion constitutes at least part of the inherited biological predisposition towards the development of depression with somatization predicted by Freud. Furthermore, although our present findings require additional validation in varied groups of patients, our preliminary data suggest that a small number of specific somatic-related questions, inquiring for symptoms that likely would be instantly recognizable by Briquet and Freud, can be constructed into a valid screening instrument for cases at high risk for having a component of energy metabolism in their pathogenesis, or "mitosomatic" illness.

## Abbreviations

KSP = Karolinska Scales of Personality; DSM = Diagnostic and Statistical Manual; ATP = Adenosine triphosphate; ADHD = Attention Deficit Hyperactivity Disorder; mtDNA = Mitochondrial DNA; P = Probability.

## Competing interests

The author(s) declare that they have no competing interests.

## Authors' contributions

AG conducted the Swedish study and RB conducted the American study. Both authors contributed to the study design, drafted the manuscript, and read and approved the final version.
